# Mood symptoms, neurodevelopmental traits, and their contributory factors in X‐linked ichthyosis, ichthyosis vulgaris and psoriasis

**DOI:** 10.1111/ced.15116

**Published:** 2022-03-04

**Authors:** Georgina H. Wren, Trevor Humby, Andrew R. Thompson, William Davies

**Affiliations:** ^1^ School of Psychology Cardiff University Cardiff UK; ^2^ Neuroscience and Mental Health Research Institute Cardiff University Cardiff UK; ^3^ South Wales Clinical Psychology Doctoral Programme Cardiff and Vale University Health Board Cardiff UK; ^4^ MRC Centre for Neuropsychiatric Genetics and Genomics and Division of Psychological Medicine and Clinical Neurosciences School of Medicine Cardiff University Cardiff UK

## Abstract

**Background:**

High rates of adverse mood/neurodevelopmental traits are seen in multiple dermatological conditions, and can significantly affect patient quality of life. Understanding the sex‐specific nature, magnitude, impact and basis of such traits in lesser‐studied conditions like ichthyosis, is important for developing effective interventions.

**Aim:**

To quantify and compare relevant psychological traits in men with X‐linked ichthyosis (XLI, *n* = 54) or in XLI carrier women (*n* = 83) and in patients with ichthyosis vulgaris (IV, men *n* = 23, women *n* = 59) or psoriasis (men *n* = 30, women *n* = 122), and to identify factors self‐reported to contribute most towards depressive, anxious and irritable phenotypes.

**Methods:**

Participants recruited via relevant charities or social media completed an online survey of established questionnaires. Data were analysed by sex and skin condition, and compared with general population data.

**Results:**

Compared with the general population, there was a higher rate of lifetime prevalence of mood disorder diagnoses across all groups and of neurodevelopmental disorder diagnoses in the XLI groups. The groups exhibited similarly significant elevations in recent mood symptoms (Cohen *d* statistic 0.95–1.28, *P* < 0.001) and neurodevelopmental traits (*d* = 0.31–0.91, *P* < 0.05) compared with general population controls, and self‐reported moderate effects on quality of life and stigmatization. There were strong positive associations between neurodevelopmental traits and recent mood symptoms (*r* > 0.47, *P* < 0.01), and between feelings of stigmatization and quality of life, particularly in men. Numerous factors were identified as contributing significantly to mood symptoms in a condition or sex‐specific, or condition or sex‐independent, manner.

**Conclusion:**

We found that individuals with XLI, IV or psoriasis show higher levels of mood disorder diagnoses and symptoms than matched general population controls, and that the prevalence and severity of these is similar across conditions. We also identified a number of factors potentially conferring either general or condition‐specific risk of adverse mood symptoms in the three skin conditions, which could be targeted clinically and/or through education programmes. In clinical practice, recognizing mood/neurodevelopmental problems in ichthyosis and psoriasis, and addressing the predisposing factors identified by this study should benefit the mental health of affected individuals.

## Graphical abstract

In this study, we have quantified mood and neurodevelopmental traits across three dermatological conditions (X‐linked ichthyosis, ichthyosis vulgaris and psoriasis) and across the sexes. We show high levels of mood and neurodevelopmental traits in all three conditions, and identify potential contributory factors amenable to clinical intervention.

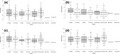

## Introduction

Dermatological conditions are associated with elevated rates of mental health problems including mood disorders.^1^, ^2^, ^3^ For many of these conditions, there is a lack of research into the nature and causes of these psychological difficulties, and access to psychological support is severely limited.^4^ Mood problems associated with skin conditions may have multiple causes, including stigmatization or bullying,^5^ negative impacts upon education, employment or social life,^6^ pain/itch,^7^ associated sleep problems,^8^, ^9^ a need to source and apply medications,^10^, ^11^ and comorbid physical ill‐health,^12^, ^13^ and such problems can significantly affect patient quality of life (QoL).^14^

Psychodermatological research has often focused on comparing mental health outcomes in specific conditions with those of unaffected controls, using relatively small, clinically ascertained samples originating from a single geographical region, and using a limited range of measures. There is therefore a need to compare psychological symptoms in patients vs. controls and across different skin conditions, using multiple reliable measures and in large and geographically diverse samples; moreover, given the sex‐biased nature of many skin conditions^15^ and psychological phenomena (e.g. depression and anxiety^16^), stratifying psychodermatological outcomes by sex is warranted.

The psychological correlates of common dermatological conditions such as eczema and psoriasis have been investigated intensively,^17^, ^18^ and may be used as appropriate comparators for other, less well‐studied conditions. Psoriasis is a chronic, inflammatory, multiorgan disease affecting 2% of UK adults, and is characterized by demarcated, salmon‐coloured plaques and extracutaneous manifestations such as arthritis and diabetes.^19^, ^20^ Psoriasis has been associated with a substantially increased risk of depression or anxiety disorders^21^, ^22^ and neurodevelopmental conditions, notably autism spectrum disorder.^23^, ^24^ The correlates of rarer skin conditions such as the inherited ichthyoses, which are characterized by abnormal keratinization,^25^ are comparatively less well‐defined, although there is recent evidence that across the ichthyoses, rates of psychological distress are raised while QoL is diminished.^26^, ^27^ Qualitative data have suggested that QoL impairment in inherited ichthyoses may be due to a combination of factors, including physical health, daily life issues, and relationships with oneself and others.^28^ As the ichthyoses have differences in aetiology, severity and epidemiology, there is a need to study each separately.

The most common inherited ichthyosis, ichthyosis vulgaris (IV), affects around 1 in 100–250 of the northern European white population and has a complex genetic aetiology often involving the filaggrin (*FLG*) gene.^29^, ^30^ New evidence suggests an elevated rate of mood disorders/symptoms in patients with IV, together with a moderately decreased QoL and increased social rejection.^27^ X‐linked ichthyosis (XLI), a disorder caused by deletion or loss‐of‐function genetic variants around the steroid sulfatase (*STS*) gene, affects approximately 1 in 3000–6000 men.^31^ Both men with XLI and women carrying the XLI‐associated genetic variants exhibit elevated rates of mood and neurodevelopmental disorders (and associated traits) compared with sex‐matched controls.^32^, ^33^, ^34^, ^35^, ^36^ Potentially, adult mood symptoms may be related to deficits in executive function arising developmentally.^37^, ^38^

Using a large sample of men and women participants with confirmed diagnoses of XLI (or carriers of the XLI‐associated genetic variant), IV or psoriasis recruited from around the world and tested via established online survey methodology,^39^ we investigated: (i) mood/neurodevelopmental disorders and associated traits in our six study groups compared with the best‐available matched general population samples, (ii) mood/neurodevelopmental disorders and associated traits compared across the six groups and (iii) factors self‐reported to contribute most substantially towards mood (depressive, anxiety and irritability) symptoms across the six groups. A key aim of the study was to identify the best target(s) for intervention/therapy to minimize mood problems associated with the ichthyoses and psoriasis.

## Methods

The study was approved by Cardiff University School of Psychology Research Ethics Committee, and all patients provided informed consent.

### Participant recruitment and survey completion

Adults (age > 18 years) with a past diagnosis of XLI, IV or psoriasis, or with confirmed carrier status for an XLI‐associated genetic variant, were recruited from around the world via past contact, charities, social media patient support groups and other social media sites (e.g. Twitter) and directed to an online survey. Anonymous responses were returned to the research team during the period 8 January to 27 April 2021.

### Survey structure

Participants initially provided the following demographic information: age, country of residence, ethnicity and highest education level obtained, as well as information regarding the basis of their dermatological diagnosis or how their carrier status was determined. They then provided information about any previous neurodevelopmental/mood disorder diagnoses, before rating their skin condition on average throughout their life (women XLI carriers excluded) using sample clinical images from the Congenital Ichthyoses Severity Index (CISI)^40^ (range 2–8) or the Psoriasis Area and Severity Index (PASI)^41^ (range 3–15).

Subsequently, participants completed a series of well‐characterized psychological measures (scoring details in Supplementary Data S1): (i) the Kessler Psychological Distress Scale (K10) assessing recent depression/anxiety‐related traits,^42^ (ii) the Adult Attention Deficit Hyperactivity Disorder (ADHD) Self‐Report Scale (ASRS) V1.1 assessing ADHD‐related traits,^43^ (iii) the Short Autism Spectrum Quotient (AQ10) assessing traits associated with autism spectrum disorders,^44^ (iv) the Brief Irritability Test (BITE) assessing irritability traits,^45^ (v) the Dermatology Life Quality Index (DLQI) assessing dermatology‐specific QoL^14^ and (vi) the Feelings of Stigmatization Questionnaire (FSQ) assessing levels of skin disease‐related stigmatization^46^ (note that lower scores indicate higher levels of perceived stigma). As women XLI carriers lack significant skin pathology, they did not complete the DLQI or FSQ. A composite ‘neurodevelopmental trait score’ (NTS) to which ASRS and AQ10 scores contributed equally was also calculated (range 0–144). Finally, participants completed an 11‐point sliding response scale (0 = not at all, 10 = very much) regarding the perceived impact of 19 factors (Supplementary Table S1) on depressive, anxious and irritability traits.

### Data analysis and statistics

Questionnaires were analysed as previously described in the literature. In the rare circumstance of participants not answering specific questions (< 2% of all responses), mean group values were imputed. Summary data are reported as percentages, mean ± SD (if data normally distributed) or median with 95% CI (if data not normally distributed). Between‐group data were compared by unpaired *t*‐test, one‐way ANOVA or Kruskal–Wallis *H*‐test, depending upon their distribution. Data from each experimental group were compared with the best‐available matched general population control samples using Welch *t*‐test with the degrees of freedom calculated using the Welch–Satterthwaite equation. *P* values were calculated from the resultant *t* statistic, and degrees of freedom and Cohen *d* statistic is presented as a measure of effect size. For two‐tailed analyses with α = 0.05 and a general population sample > 10 times that of the experimental group, ≥ 35 participants per group provided > 80% power to reliably detect a medium‐sized effect (Cohen *d* = 0.5). Subsequently, experimental groups were directly compared using unpaired *t*‐test or ANOVA with main factors of sex (men/women) and condition (XLI, IV, psoriasis). An overall sample of ≥ 235 participants enabled reliable detection of main and interaction effects of small–medium effect size (partial η^2^ = 0.04) with > 80% power (α = 0.05). Boxplots were produced using BoxPlotR software.^47^ Pearson and Spearman tests of correlation were performed for normally and non‐normally distributed data, respectively, and hierarchical linear regression was performed to determine the variance explained by relevant factors on recent mood and QoL. *P* < 0.05 was considered statistically significant. Finally, mean scores for each factor potentially predisposing to aspects of mood for each experimental group were calculated and ranked.

## Results

### Participants

We recruited 371 participants: 54 men with XLI, 83 women who were carriers of XLI‐associated genetic variants, 23 men with IV, 59 women with IV, 30 men with psoriasis and 122 women with psoriasis. Participants were predominantly diagnosed by a medical professional taking into account family history and with a confirmatory biochemical/genetic test where appropriate. Groups were closely matched for age (medians 40–50 years, *H*_(5)_ = 5.11, *P* = 0.40), country of residence (74%–87% UK/North America), ethnicity (74%–91% white European descent) and highest academic qualification (63%–85% college, university or postgraduate education). The mean self‐reported skin severity was similar (moderate) across groups (40%–53% of maximum score on CISI/PASI).

### Psychological diagnoses

We summarized the prevalence of previous diagnoses of mood or neurodevelopmental condition across the six groups and compared this with lifetime diagnostic prevalence in the general population (Table 1). The prevalence of depression diagnoses across all groups was noticeably higher than in the general population and, interestingly, was highest in XLI carrier women, even though these individuals display mild, if any, skin symptoms. The prevalence of anxiety disorder diagnoses was only substantially higher than the general population prevalence for both men with XLI and XLI carrier women, and for men with IV. The prevalence of previous bipolar disorder diagnoses was slightly higher in women with IV group than in the general population, but no similar effect was seen for the other groups. The prevalence of neurodevelopmental disorder diagnoses was substantially higher than the general population prevalence in both men with XLI and women who were carriers of XLI, but not in the other groups. The prevalence of dyslexia diagnoses was low and comparable with the general population rate, across all groups.

**Table 1:** Prevalence of mood and neurodevelopmental disorders in the general population and in the six experimental groups.

Prevalence	Depression	Bipolar disorder	Anxiety disorder (including OCD)	Autism spectrum condition	ADHD/ADD	Dyslexia
General population, %						
Male	19^54^	–	14^55^	1^56^	5^57^	–
Female	27^54^	–	23^55^	0.1^56^	4^57^	–
Both sexes	–	2^58^	–	–		2–11^59^
XLI, %						
Male	31	2	31	10	19	6
Female carrier	45	1	30	1	10	3
IV, %						
Male	35	4	22	0	4	0
Female	37	7	19	0	7	3
Psoriasis, %						
Male	40	0	10	0	0	0
Female	33	1	25	0	2	0

ADD, attention deficit disorder; ADHD, attention deficit hyperactivity disorder; IV, ichthyosis vulgaris; OCD, obsessive–compulsive disorder; XLI, X‐linked ichthyosis.

### Comparison of mood/neurodevelopmental traits with general population samples and between groups

**Figure 1:**
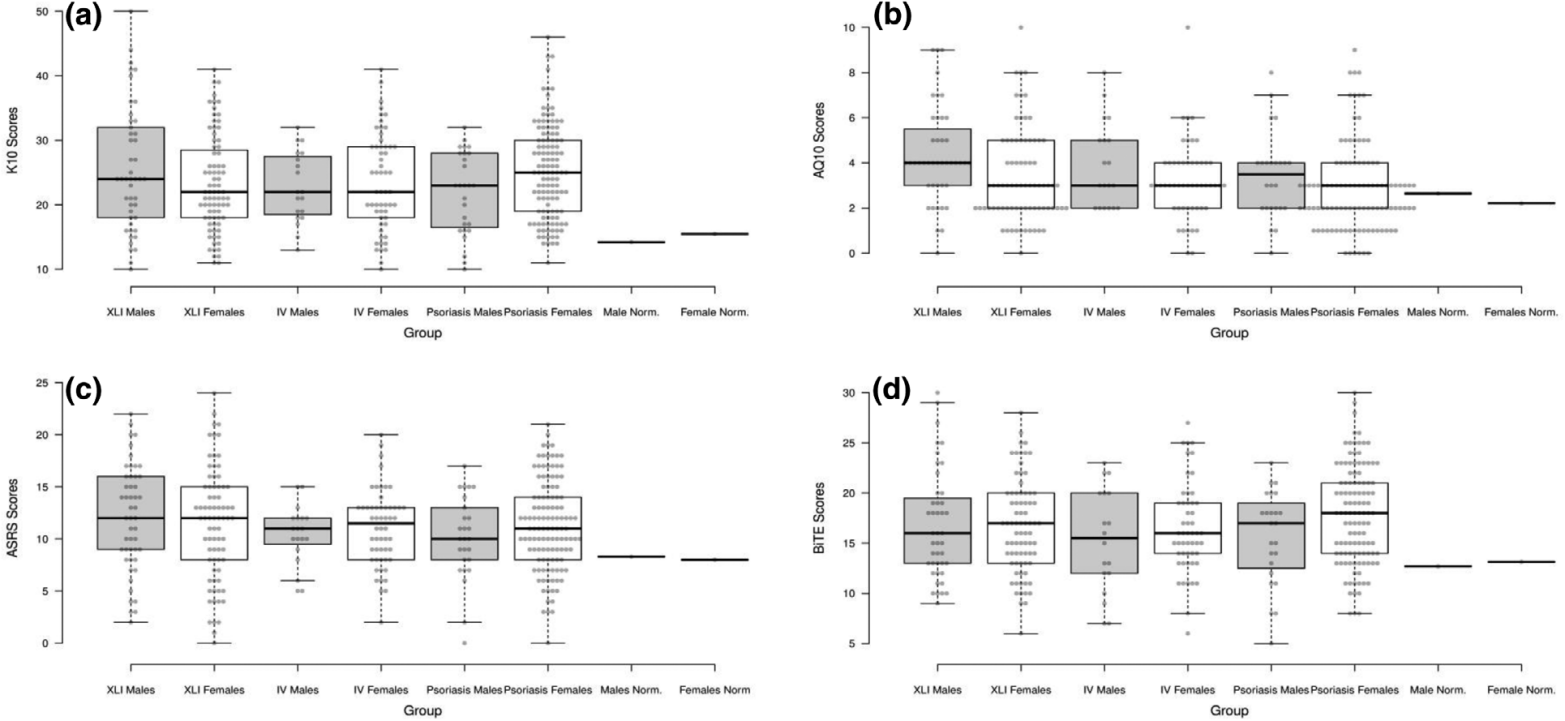
Boxplots showing scores on (a) the Kessler psychological distress scale (K10), (b) the short Autism Spectrum Quotient (AQ10), (c) the Adult Attention Deficit Hyperactivity Disorder Self‐report Scale (ASRS) Part A and (d) the Brief Irritability Test (BITE) across all six experimental groups. Centre lines show the medians, box limits indicate the 25th and 75th percentiles as determined by *R* software, and whiskers extend 1.5 times the interquartile range from the 25th and 75th percentiles. Mean values for general population normative samples for both men and women are shown for comparison. IV, ichthyosis vulgaris; XLI, X‐linked ichthyosis.

K10 scores were substantially and significantly higher in all experimental groups (Cohen *d* = 0.95–1.28, *P* < 0.001) than in the general population [K10 controls (unscreened community‐recruited Australian adult women (*n* = 882, 35–44 years) and men (*n* = 566, 45–54 years)^48^]; average scores in our experimental groups were consistent with having a mild to moderate mental disorder (Fig. 1a, Supplementary Table S2). Theoretically, given that the current survey was administered during the COVID‐19 pandemic, scores on the K10 measure might have been inflated in our experimental groups, and this might explain the difference from general population controls tested prior to the pandemic. However, when compared with previously obtained K10 scores in male XLI^36^ and female XLI carrier^33^ participants, current scores in these groups were not significantly different and were actually slightly lower on average (men: 28.4 ± 9.3 vs. 25.2 ± 9.8, respectively, *U* = 886.5, *P* = 0.11; women: 23.6 ± 8.6 vs. 23.3 ± 7.2 respectively, *U* = 3531.5, *P* = 0.96). Given these data and as there is no reason to suspect that COVID‐19 affects mood symptoms differentially across skin conditions, we believe that the elevated K10 scores across groups are not a consequence of pandemic‐related issues. Scores did not differ by condition (*F*_(2,345)_ = 0.56, *P* = 0.57) or sex (*F*_(1,345)_ = 0.49, *P* = 0.49). There was a significant condition × sex interaction (*F*_(2,345)_ = 3.28, *P* < 0.04), but no *post hoc* pairwise comparisons were significant (Fig. 1a).

AQ10 scores across all groups were, moderately and significantly, higher (Cohen *d* = 0.31–0.70, *P* < 0.05) than those observed in a previously reported^49^ unscreened adult (45–64 years) sample of 10 796 women and 7904 men recruited through a general population health survey in Sweden (Fig. 1b, Supplementary Table S2). As expected, AQ10 scores were higher in men than in women (effect of sex: *F*_(1,319)_ = 7.44, *P* < 0.01), but did not differ by condition (*F*_(2,319)_ = 2.86, *P* = 0.06) or as a function of condition × sex (*F*_(2,319)_ = 0.37, *P* = 0.69) (Fig. 1b).

Scores on Part A of the ASRS were also significantly higher (Cohen *d* = 0.59–0.91, *P* < 0.05) in all of our experimental groups than in a previously reported group of general population controls^50^ [unscreened community‐recruited Australian adult (47–54 years) sample of 1098 women and 993 men] (Fig. 1c, Supplementary Table S2). Inattentive symptom scores were higher than hyperactive–impulsive symptom counts in all groups (Supplementary Table S3). ASRS scores did not differ by condition (*F*_(2,334)_ = 0.79, *P* = 0.46) or sex (*F*_(1,334)_ = 1.84, *P* = 0.18) or as a function of condition × sex (*F*_(2,334)_ = 0.51, *P* = 0.60) (Fig. 1c).

On average, all our groups scored more highly on the BITE compared with a previously reported^45^ combined sample of undergraduate students and outpatient clinic‐recruited chronic pain patients (711 women, 405 men; mean age 28 years) (Fig. 1d, Supplementary Table S2); however, formal statistical analysis was not possible. There was no significant effect of condition (*F*_(2,319)_ = 0.62, *P* = 0.54), sex (*F*_(1,319)_ = 2.78, *P* = 0.10) or condition × sex (*F*_(2,319)_ = 1.36, *P* = 0.26) (Fig. 1d).

### Quality of life and stigmatization in groups with skin conditions

**Figure 2:**
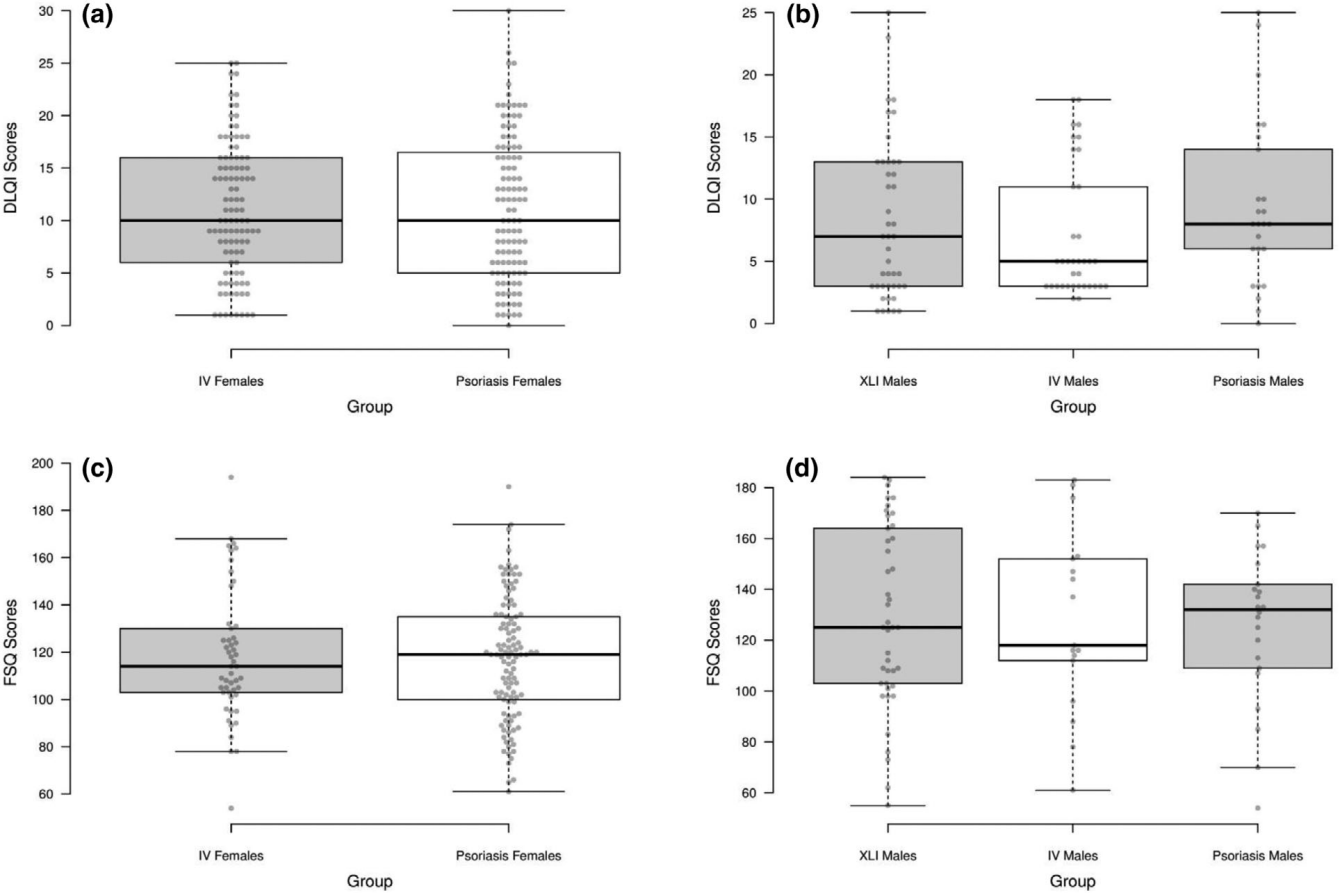
Boxplots showing (a,b) Dermatology Life Quality Index (DLQI) scores in (a) men and (b) women, and Feelings of Stigmatization Questionnaire (FSQ) scores in (c) men and (d) women. Centre lines show the medians, box limits indicate the 25th and 75th percentiles as determined by *R* software, and whiskers extend 1.5 times the interquartile range from the 25th and 75th percentiles. IV, ichthyosis vulgaris; XLI, X‐linked ichthyosis.

Women with IV or psoriasis exhibited equivalent scores (median around 10) on the DLQI (*t*_(1,161)_ = 0.13, *P* = 0.72), consistent with a moderate to large effect on QoL (Fig. 2a). Men with XLI, IV or psoriasis reported equivalent scores on the DLQI (*F*_(2,83)_ = 0.88, *P* = 0.42), which were, on average, lower than those reported by women (median 5–10), consistent with moderate effects on QoL (Fig. 2b).

Women with IV or psoriasis exhibited equivalent median scores (100–120) on the FSQ (*t*_(1,157)_ = 0.035, *P* = 0.85, Fig. 2c), and men with XLI, IV or psoriasis also exhibited similar median scores (around 125) on this measure (*F*_(2,79)_ = 0.45, *P* = 0.64, Fig. 2d).

### Relationships between skin severity and psychological measures across groups

We predicted that greater skin condition severity would be positively associated with a history of depression and adverse mood‐related traits. However, we found that a previous diagnosis of depression was not associated with significantly higher CISI/PASI scores for any of the groups (Supplementary Fig. S1). We identified weak, positive correlations between skin severity and recent adverse mood symptoms in women with IV (*r*_s_ = 0.30, *P* = 0.026) or psoriasis (*r*_s_ = 0.34, *P* < 0.001), but not in men with XLI (*r*_s_ = 0.04, *P* = 0.80), IV (*r*_s_ = 0.21, *P* = 0.40) or psoriasis (*r*_s_ = 0.26, *P* = 0.19).

In men with XLI, IV or psoriasis, the NTS explained 34%–40% of the variance within the K10 score after adjusting for skin severity, whereas in women with IV or psoriasis, this rate was lower (7%–12%). The skin severity score explained just 1%–9% (median 3%) of the variance within the K10 score after adjusting for the NTS across all five groups. After adjusting for both NTS and skin severity, the K10 score explained 14%–27% of the variance in DLQI scores in men with XLI and in both men and women with psoriasis, but < 1% of the variance in DLQI scores in both men and women with IV.

Skin severity did not seem to correlate strongly with feelings of stigmatization in any group, with a significant relationship being observed only for women with psoriasis (*r* = −0.21, *P* = 0.030; all other groups, *r* = −0.17 to 0.35, *P* > 0.12). After adjusting for NTS and skin severity scores, FSQ scores explained 14%–18% of the variance in K10 scores in both men and women with psoriasis groups, 10% in the men with XLI and 3% in both men and women with IV. After adjusting for NTS and skin severity scores, FSQ scores explained 32%–40% of the variance in DLQI scores in men with XLI, IV or psoriasis, and slightly less (17%–25%) in women with IV or psoriasis. Correlations between the various psychological measures are reported in Supplementary Tables S4 and S5.

### Factors influencing adult mood

**Figure 3:**
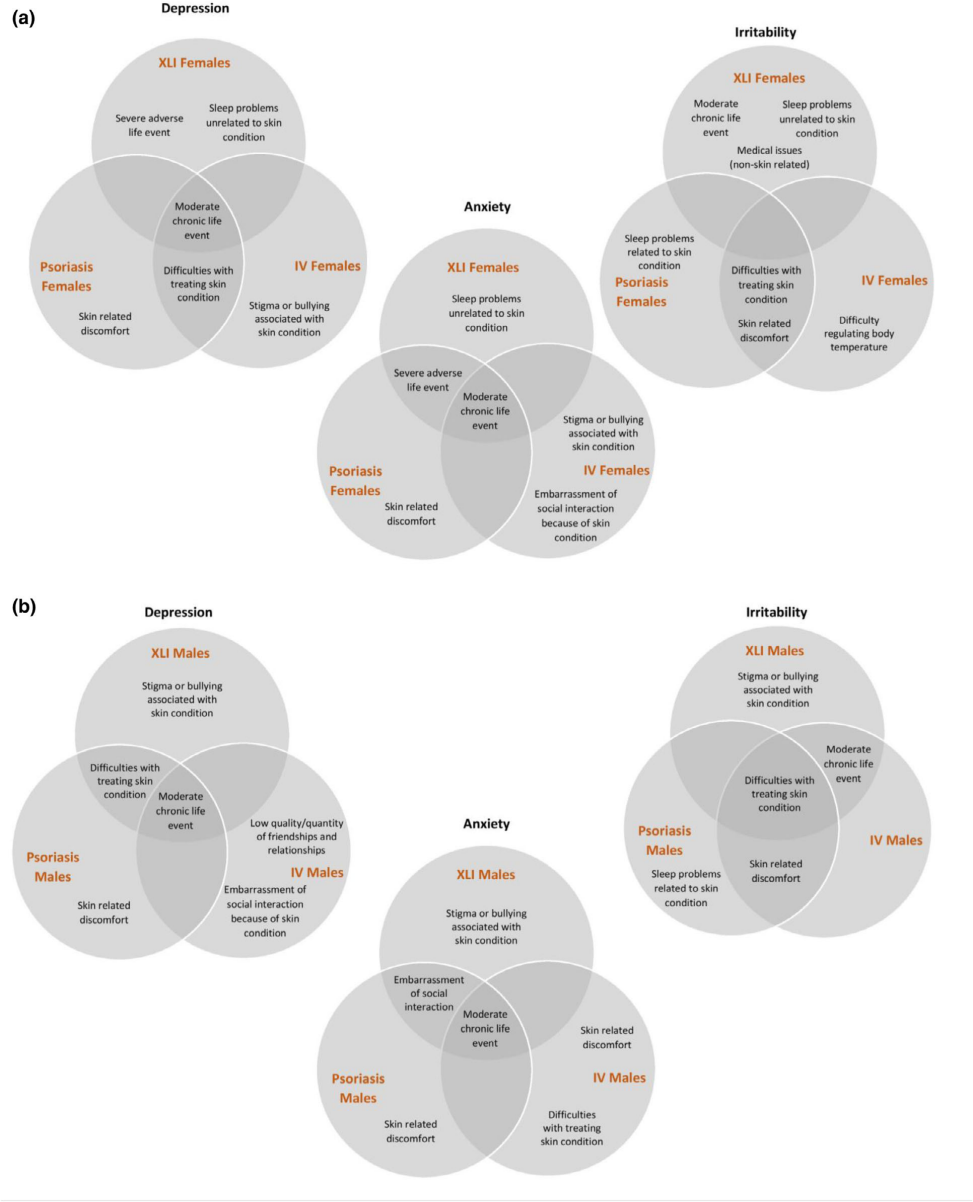
Venn diagrams illustrating the three factors self‐reported to be most strongly associated with aspects of mood (depression, anxiety, irritability) across the experimental groups of (a) women and (b) men. IV, ichthyosis vulgaris; XLI, X‐linked ichthyosis. [Colour figure can be viewed at wileyonlinelibrary.com]

The top three self‐reported factors influencing aspects of mood for each group are illustrated in Fig. 3, with the ranking for all 19 factors shown in Supplementary Figs S2–S7. A qualitative analysis of free‐text comments by participants is presented in Supplementary Table S6.

In women, ‘skin‐related stigma or bullying’, ‘embarrassment of social interaction’ and ‘difficulty regulating body temperature’ were highlighted only by the IV patient group as particular issues of concern. ‘Sleep problems related to the skin condition’ were highlighted as a main issue of concern for women with psoriasis, but not for women with IV. Common concerns in women with IV and women with psoriasis were ‘skin‐related discomfort’ and ‘difficulties with treating the skin condition’. The main perceived drivers of mood problems in women who were carriers of XLI‐associated genetic variants were adverse life events, sleep problems and medical issues.

In men, ‘stigma or bullying associated with skin condition’ was strongly perceived to be a causal factor for depressive, anxious and irritable traits only for XLI, whereas ‘low quality or quantity of friendships or relationships’ was reported to strongly contribute to depressive symptoms only for IV, and ‘sleep problems due to the skin condition’ were perceived to be strongly associated with a mood symptom (irritability) in psoriasis. The following factors were perceived by men as contributing to ≥ 1 mood symptoms in ≥ 2 of the disease groups: ‘difficulties with treating the skin condition’, ‘skin‐related discomfort’ and ‘embarrassment of social interaction because of the skin condition’.

## Discussion

We investigated adult mood/neurodevelopmental traits along with their causes and consequences in two skin conditions poorly characterized in terms of their psychological correlates (XLI and IV) and in a better‐studied comparator condition (psoriasis), using a large online sample split by sex. Importantly, our experimental groups were closely matched for demographic factors and skin severity, so any group‐specific psychological effects were unlikely to have been attributable to these variables.

Our first main finding was an increase in depression diagnoses across disorders and, consistent with this, a similarly sized (medium to large) increase in number of recent adverse mood/irritability symptoms across disorders compared with general population controls; the latter finding did not appear to be COVID‐19‐related. Elevated rates of depression have been reported in psoriasis,^21^, ^22^ XLI^33^, ^36^ and IV,^27^ but the three conditions have never previously been directly compared. Our study emphasizes that adverse mood symptoms in men with XLI, women who are XLI carriers, and both men and women with IV can be substantial and of a comparable magnitude to those seen in psoriasis. All three disorders were also associated with a moderate to large effect on QoL and high feelings of stigmatization.

Our second substantive finding, replicating previous work,^33^, ^34^, ^35^, ^36^ was that men with XLI and women who were carriers of XLI‐associated genetic variants exhibited significantly higher rates of neurodevelopmental disorder diagnoses than general population controls; the high rates of mood and neurodevelopmental traits seen in XLI carrier women who do not exhibit skin pathology suggest that deficiency of *STS* or adjacent genes elicits a neurobehavioural effect independent of that associated with the skin condition. We also found that across all three conditions, self‐reported levels of ADHD (particularly inattentive) and autism‐related traits were equal, and they were moderately elevated compared with general population levels. These data add to the growing body of evidence that neurodevelopmental conditions/traits are elevated in multiple skin conditions,^51^, ^52^ and suggest the possibility that some individuals with IV or psoriasis exhibiting high levels of neurodevelopmental traits are not being appropriately diagnosed. The co‐occurrence of dermatological and psychological phenotypes indicates not only the need for further research to investigate common links, but also a need for greater multidisciplinary involvement in patient care. We recognize potential generalizability limitations given that our samples were drawn from multiple sources and from community rather than clinical settings; however, the fact that ichthyosis is a comparatively rare condition necessitated such an approach. Nevertheless, respondents self‐reported an appropriate dermatological diagnosis, data were consistent with previous questionnaire‐based findings in XLI and internal consistency across questionnaires as indexed by Cronbach α was generally high (> 0.8) and comparable across groups (Supplementary Table S7).

We have begun to explore factors associated with and potentially causal for the elevated mood symptoms seen in the three skin conditions described here. Skin severity scores did not differ between individuals who had been diagnosed with depression and those who had not, and there were only weak associations noted between skin severity and recent adverse mood symptoms/feelings of stigmatization in women. These findings suggest the possibility that increased skin condition severity only confers a slight increase in risk of exhibiting mood symptoms or feelings of stigmatization, over and above being affected by a skin condition *per se*. We noted a strong positive relationship between NTS and adverse mood symptoms, particularly in men, and this pattern of data mirrors that obtained in other studies in general population samples.^50^ There are three possible explanations for this relationship: (i) high levels of neurodevelopmental problems confer a later risk of mood problems, (ii) high levels of mood problems elicit high levels of neurodevelopmental disorder‐associated traits (or phenocopies thereof), e.g. through impairing sleep quality and subsequent executive function^53^ and (iii) the biology of the skin condition (e.g. genetic factors, skin permeability, inflammation) affects neurodevelopment and mood‐related processes via dissociable mechanisms. Unexpectedly, no group self‐reported neurodevelopmental traits and the potential life challenges associated with these as being a major contributory factor towards their mood symptoms. Therefore, options (ii) and/or (iii) seem more likely explanations for the observed relationship.

In men with XLI and both men and women with psoriasis, recent mood symptoms were somewhat related to QoL; however, this relationship was not apparent for either men or women with IV group, suggesting that QoL in this condition is less strongly linked to mood. Across groups, FSQ scores did not strongly predict recent mood; potentially, being stigmatized may contribute towards mood across the lifespan, but not necessarily at the timepoint we assessed. However, stigmatization did predict perceived QoL to some extent, particularly in men.

Our study highlights both general and condition‐specific factors that patients perceive as contributing towards mood symptoms and that might be targeted to mitigate adverse psychological effects. General predisposing factors identified included skin‐related discomfort and difficulties with treating the skin condition; thus patients with XLI, IV and psoriasis should routinely be advised on where to source treatments and how/when to apply these with a view to minimizing pain and inconvenience. Stigma and bullying appears to be an issue for men with XLI and women with IV, whereas low quality or quantity of friendships appears to be an issue in men with IV, thus promoting resilience/coping strategies in these groups through psychological therapies, as well as educating society about medical conditions affecting appearance should be a priority. Practical advice from dermatologists regarding optimal strategies for regulating body temperature and for night‐time skin management, may alleviate irritability in women with IV and in patients with psoriasis, respectively.

## Conclusion

We have shown that rates of mood disorder diagnoses and levels of associated traits are similar across three dermatological conditions, and are significantly higher than in the general population. We have also identified general, and condition‐specific, factors that may influence vulnerability to adverse mood symptoms and that may be amenable to clinical intervention and educational strategies.

## Conflict of interest

ART has acted as an unpaid clinical psychology advisor to the All Party Parliamentary Group on Skin Disease, has received research support from Pfizer and has received honorariums for educational presentations from pharmaceutical companies including Novartis and UCB. The other authors have no conflicts of interest to declare.

**What's already known about this topic?**

• Adverse mood symptoms are common in dermatological conditions.
• Some conditions, including psoriasis and XLI also appear to be associated with an increased risk of neurodevelopmental disorders and associated traits.
• Mood and neurodevelopmental disorder diagnoses/traits in IV have not been studied in any depth and XLI, IV and psoriasis have not previously been compared in relation to these areas of functioning.

**What does this study add?**

• Our study quantifies multiple self‐reported mood and neurodevelopmental disorder‐related measures in individuals with XLI (or carrier women), psoriasis or IV, allowing comparison across these conditions and with general population controls.
• It demonstrates high, comparable and largely sex‐independent levels of adverse mood symptoms and neurodevelopmental traits across conditions, which are strongly correlated in men.
• It also identifies common and condition‐specific, factors that contribute to mood and that are amenable to intervention.
• There is a greater need for routine assessment of mood disturbance, neurodevelopmental problems and stigma in patients with ichthyosis or psoriasis.
• It is also important to assess level of social support in the form of friendships in patients with ichthyosis (particularly in men with IV) or psoriasis.
• Where issues are identified, there needs to be clear pathways for providing access to psychological intervention and support.

## Supporting information


**Data S1.** Appendix S1: Psychological scales and their scoring.
**Figure S1.** Dot plots showing the relationship between skin severity as indexed by self‐report based upon Congenital Ichthyosis Severity Index or Psoriasis Area and Severity Index (as appropriate) and a diagnosis of depression across the groups.
**Figures S2–S7.** Ranking of factors (scored from 0 to 10) potentially predisposing to adult mood symptoms (depression, anxiety and irritability in all six groups.
**Table S1.** Factors that could potentially predispose to adult mood symptoms.
**Table S2.** Comparison of the scores on the Kessler Psychological Distress Scale (K10), the Adult Attention Deficit Hyperactivity Disorder Self‐Report Scale (ASRS) Part A, the Short Autism Spectrum Quotient (AQ10) and the Brief Irritability Test (BITE) vs. those on normative samples across the six groups.
**Table S3.** Inattentive vs. hyperactive–impulsive symptom counts across the Adult Attention Deficit Hyperactivity Disorder Self‐Report Scale (ASRS) for all six groups.
**Table S4**. Relationship between recent mood, neurodevelopmental traits and irritability across the groups.
**Table S5.** Relationship between recent mood, quality of life and feelings of stigmatization across the groups.
**Table S6.** A qualitative deductive content analysis of free‐text comments relating to factors underlying mood symptoms across the lifespan across the three conditions.
**Table S7.** Internal consistency as indexed by Cronbach α across questionnaires and across experimental groups.
