# Supplementary material for: Mood symptoms, neurodevelopmental traits, and their contributory factors in X‐linked ichthyosis, ichthyosis vulgaris and psoriasis
Source: Clin Exp Dermatol. 2022 Mar 4;47(6):1097–108. doi: 10.1111/ced.15116 (PMC9314151; doi:10.1111/ced.15116)
Supplement: Supplementary file 1 — Data S1. Appendix S1: Psychological scales and their scoring. Figure S1. Dot plots showing the relationship between skin severity as indexed by self‐report based upon Congenital Ichthyosis Severity Index or Psoriasis Area and Severity Index (as appropriate) and a diagnosis of depression across the groups. Figures S2–S7. Ranking of factors (scored from 0 to 10) potentially predisposing to adult mood symptoms (depression, anxiety and irritability in all six groups. Table S1. Factors that could potentially predispose to adult mood symptoms. Table S2. Comparison of the scores on the Kessler Psychological Distress Scale (K10), the Adult Attention Deficit Hyperactivity Disorder Self‐Report Scale (ASRS) Part A, the Short Autism Spectrum Quotient (AQ10) and the Brief Irritability Test (BITE) vs. those on normative samples across the six groups. Table S3. Inattentive vs. hyperactive–impulsive symptom counts across the Adult Attention Deficit Hyperactivity Disorder Self‐Report Scale (ASRS) for all six groups. Table S4. Relationship between recent mood, neurodevelopmental traits and irritability across the groups. Table S5. Relationship between recent mood, quality of life and feelings of stigmatization across the groups. Table S6. A qualitative deductive content analysis of free‐text comments relating to factors underlying mood symptoms across the lifespan across the three conditions. Table S7. Internal consistency as indexed by Cronbach α across questionnaires and across experimental groups. [file CED-47-1097-s001.docx]

**Appendix S1:** Psychological scales and their scoring

**Kessler Psychological Distress Scale (K10)**

A five-point 10-item Likert-scale questionnaire assessing recent depression or anxiety-related traits. K10 items were scored from 1–5 (total score range 10–50) with a score ≥20 being consistent with significant psychological distress

**Adult ADHD Self-Report Scale version 1.1 (ASRS v1.1)**

A five-point response (scored 0-4) 18-item Likert-scale screening questionnaire assessing recent attentional (nine items) and hyperactive-impulsive (nine items) traits based on diagnostic criteria for ADHD; total scores from the Part A screener (0-24) and overall questionnaire (0-72) were calculated, as were hyperactive-impulsive and inattentive symptom scores (0-9)

**Short Autism Spectrum Quotient (AQ10)**

A four point 10-item Likert-scale questionnaire, assessing behavioural traits associated with autism spectrum disorders; items endorsed as being consistent with autism-related traits were with scored with 1 point (total score range 0-10) with a score of ≥6 being consistent with a referral for a comprehensive autism assessment

**Brief Irritability Test (BITE)**

A six-point 5-item Likert-scale questionnaire assessing irritability traits. BITE items were scored from 1-6 (total score range 5-30), with a greater score associated with increased irritability

**Dermatology Life Quality Index (DLQI)**

A four-point 10-item Likert-scale questionnaire assessing dermatology-specific quality of life. DLQI items were scored from 0-3 (total score range 0-30), with a higher score associated with a greater impairment on quality of life

**Feelings of Stigmatisation Questionnaire (FSQ)**

A six-point, 33-item Likert-scale questionnaire assessing levels of skin disease-related stigmatisation. FSQ items were scored from 1-6 (total score range 33-198), with lower scores indicative of higher levels of perceived stigma.

**Table S1:** Factors that could potentially predispose to adult mood symptoms

| Moderate chronic life event (relationship difficulties/divorce, chronic illness, work-related stress etc.) |
| --- |
| Low quality/quantity of friendships and relationships |
| Embarrassment of social interaction because of your skin condition |
| Pain, discomfort or itching associated with your skin condition |
| Difficulties or frustration associated with treating your skin condition (e.g. regularly having to source and apply moisturizer) |
| Stigma or bullying associated with your skin condition |
| Sleep problems unrelated to your skin condition |
| Severe adverse life event (bereavement, life-threatening illness etc.) |
| Educational, work or social challenges due to finding it hard to pay attention e.g. getting distracted, not finishing work on time, not listening to instructions |
| Medical issues (non-skin related) |
| Stigma or bullying due to being impulsive e.g. making rash decisions, interrupting when people are talking, doing things without thinking of the consequences etc. |
| Stigma or bullying due to finding it hard to pay attention e.g. getting distracted, not finishing work on time, not listening to instructions |
| Educational, work or social challenges due to difficulties with social interaction e.g. not understanding another person's point of view or social norms, finding it difficult to follow conversations. not understanding humour or sarcasm etc. |
| Difficulty regulating body temperature/sweating |
| Educational, work or social challenges due to being impulsive e.g. making rash decisions, interrupting when people are talking, doing things without thinking of the consequences etc. |
| Stigma or bullying due to difficulties with social interaction e.g. not understanding another person's point of view or social norms, finding it difficult to follow conversations. not understanding humour or sarcasm etc. |
| Sleep problems related to your skin condition e.g. due to excessive itchiness, night-time sweating etc. |
| Allergies |
| Stress due to having a child with a long-term medical (skin) condition **(presented to female XLI carriers only)** |

**Table S2:** Comparison of K10, ASRS Part A, AQ10 and BITE scores vs. normative samples across the six groups

|  | **K10** | **AQ10** | **ASRS (Part A only)** | **BITE** |
| --- | --- | --- | --- | --- |
| General population sample (male) | 14.2±7.1 (n=566)^1^ | 2.6±3.7 (n=7904)^2^ | 8.3±3.5 (n=993)^3^ | 12.69^4^ |
| General population sample (female) | 15.5±8.9 (n=882)^1^ | 2.2±3.1 (n=10796)^2^ | 8.0±3.33 (n=1098)^3^ | 13.13^4^ |
| XLI male | 25.2±9.8 (n=50)  t[53]=7.76, p<0.001 | 4.3±2.1 (n=46)  t[45]=5.34, p<0.001 | 12.1±5.1 (n=49)  t[50]=5.16, p<0.001 | 16.6±5.5 (n=47) |
| XLI carrier female | 23.3±7.2 (n=78)  t[99]=8.98, p<0.001 | 3.4±2.2 (n=74)  t[75]=4.62, p<0.001 | 11.6±5.3 (n=75)  t[77]=5.81, p<0.001 | 16.7±4.6 (n=74) |
| IV male | 21.5±5.5 (n=19)  t[19]=5.63, p<0.001 | 3.9±1.9 (n=19)  t[18]=2.88, p=0.010 | 10.4±2.9 (n=19)  t[19]=3.11, p=0.006 | 14.5±5.2 (n=19) |
| IV female | 23.4±7.6 (n=57)  t[66]=7.52, p<0.001 | 3.2±1.7 (n=54)  t[57]=6.04, p=0.002 | 11.1±3.7 (n=54)  t[57]=6.04, p=0.002 | 16.4±4.5 (n=52) |
| Psoriasis male | 22.7±6.4 (n=26)  t[27]=6.59, p<0.001 | 3.6±1.9 (n=23)  t[22]=2.42, p=0.024 | 10.5±4.0 (n=25)  t[24]=2.72, p=0.012 | 16.3±4.7 (n=23) |
| Psoriasis female | 25.4±7.3 (n=122)  t[174]=13.64, p<0.001 | 3.0±1.9 (n=111)  t[116]=4.32, p<0.001 | 11.5±4.3 (n=116)  t[129]=8.51, p<0.001 | 18.0±5.0 (n=111) |

^1^Slade T, Grove R, Burgess P. Kessler Psychological Distress Scale: normative data from the 2007 Australian National Survey of Mental Health and Wellbeing. *Aust N Z J Psychiatry* 2011; 45: 308-16. ^2^Lundin A, Kosidou K, Dalman C. Measuring Autism Traits in the Adult General Population with the Brief Autism-Spectrum Quotient, AQ-10: Findings from the Stockholm Public Health Cohort. *J Autism Dev Disord* 2019; **49**: 773-80. ^3^Das D, Cherbuin N, Butterworth P *et al.* A population-based study of attention deficit/hyperactivity disorder symptoms and associated impairment in middle-aged adults. *PLoS One* 2012; **7**: e31500. ^4^Holtzman S, O'Connor BP, Barata PC *et al.* The Brief Irritability Test (BITe): a measure of irritability for use among men and women. *Assessment* 2015; **22**: 101-15.

**Table S3:** Inattentive vs. hyperactive-impulsive symptom counts across ASRS for all six groups

| **Group** | Hyperactive-Impulsive Symptom Count | Inattentive Symptom Count | Statistical comparison between hyperactive-impulsive and inattentive symptom count |
| --- | --- | --- | --- |
| XLI males | 2.7±2.1 | 4.6±2.6 | t(48)=6.12, p<0.001 |
| XLI females | 2.9±2.3 | 3.2±2.6 | t(72)=5.85 p<0.001 |
| IV males | 1.9±1.9 | 4.1±2.3 | t(18)=5.21, p<0.001 |
| IV females | 2.6±2.1 | 4.1±2.5 | t(53)=4.79, p<0.001 |
| Psoriasis males | 2.5±2.5 | 3.2±2.2 | t(24)=1.48, p=0.153 |
| Psoriasis females | 3.0±2.5 | 4.2±2.7 | t(117)=4.69, p<0.001 |

**Table S4:** Relationship between recent mood, neurodevelopmental traits and irritability across groups

| **Group** |  | **Neurodevelopmental Trait Score (NTS)** | **BITE score** |
| --- | --- | --- | --- |
| XLI males | **K10 score** | r=0.615, n=47, p<0.001 | r=0.647, n=47, p<0.001 |
|  | **Neurodevelopmental Trait Score (NTS)** | - | r=0.174, n=47, p=0.241 |
| XLI females | **K10 score** | r=0.660, n=74, p<0.001 | r=0.704, n=74, p<0.001 |
|  | **Neurodevelopmental Trait Score (NTS)** | - | r=0.427, n=74, p<0.001 |
| IV males | **K10 score** | r=0.652, n=19, p=0.002 | r=0.717, n=18, p=0.001 |
|  | **Neurodevelopmental Trait Score (NTS)** | - | r=0.753, n=18, p<0.001 |
| IV females | **K10 score** | r=0.467, n=52, p<0.001 | r=0.487, n=52, p<0.001 |
|  | **Neurodevelopmental Trait Score (NTS)** | - | r=0.342, n=52, p<0.013 |
| Psoriasis males | **K10 score** | r=0.617, n=24, p<0.001 | r=0.670, n=24, p<0.001 |
|  | **Neurodevelopmental Trait Score (NTS)** | - | r=0.569, n=24, p=0.004 |
| Psoriasis females | **K10 score** | r=0.599, n=112, p<0.001 | r=0.621, n=112, p<0.001 |
|  | **Neurodevelopmental Trait Score (NTS)** | - | r=0.542, n=112, p<0.001 |

**Table S5:** Relationship between recent mood, quality of life and feelings of stigmatisation across groups

| **Group** |  | **DLQI score** | **FSQ score** |
| --- | --- | --- | --- |
| XLI males | **K10 score** | r=0.548, n=47, p<0.001 | r=-0.483, n=46, p=0.001 |
|  | **DLQI score** | - | r=-0.253, n=45, p=0.094 |
| IV males | **K10 score** | r=0.264, n=18, p=0.290 | r=-0.141, n=17, p=0.589 |
|  | **DLQI score** | - | r=-0.411, n=17, p=0.102 |
| IV females | **K10 score** | r=0.220, n=52, p=0.117 | r=-0.305, n=50, p=0.031 |
|  | **DLQI score** | - | r=-0.198, n=50, p=0.168 |
| Psoriasis males | **K10 score** | r=0.776, n=25, p<0.001 | r=-0.485, n=23, p=0.019 |
|  | **DLQI score** | - | r=-0.476, n=22, p=0.025 |
| Psoriasis females | **K10 score** | r=0.464, n=112, p<0.001 | r=-0.455, n=109, p<0.001 |
|  | **DLQI score** | - | r=-0.294, n=109, p=0.002 |

**Figure S1:** Dot plots showing the relationship between skin severity as indexed by self-report based upon CISI (ichthyosis) or PASI (psoriasis) images and a depression diagnosis in males with XLI (A), males with IV (B), females with IV (C), males with psoriasis (D) and


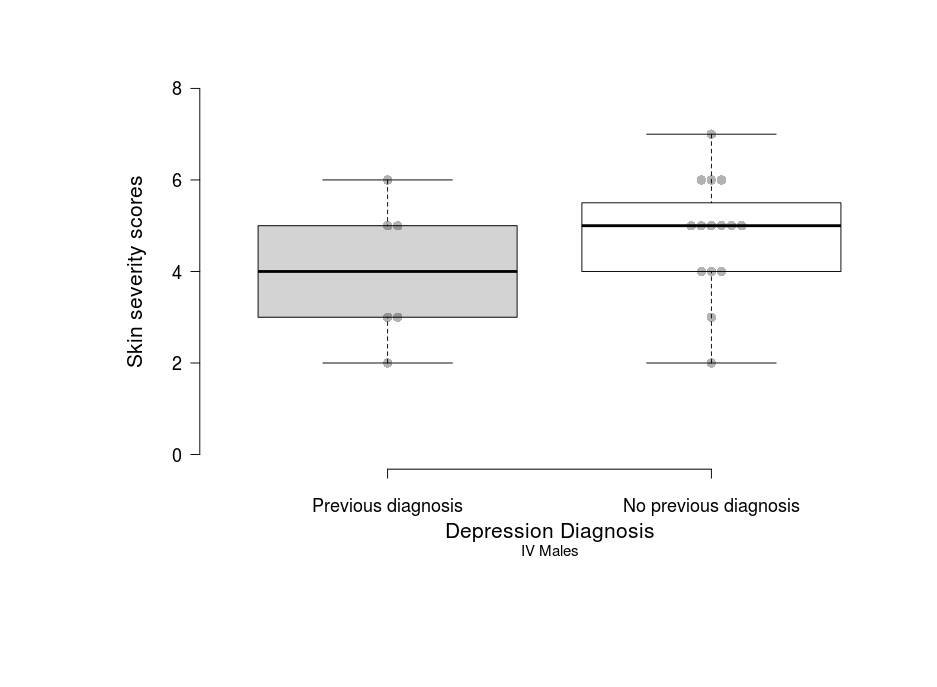

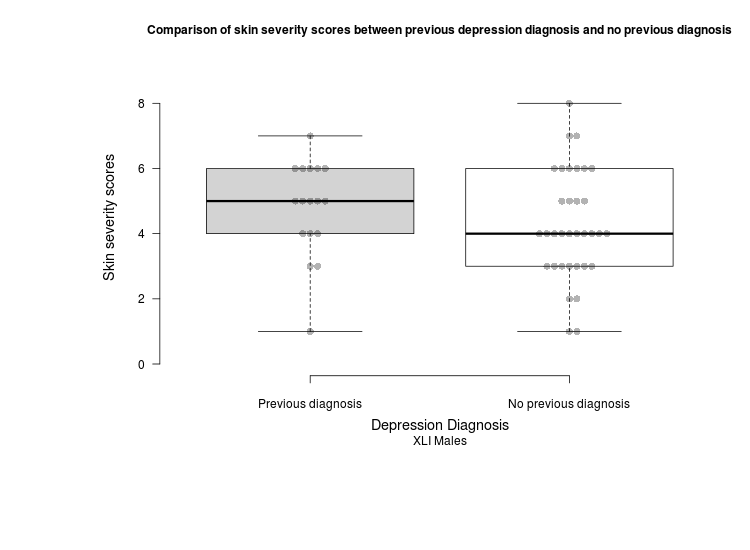
females with psoriasis (E).

**B**

**A**


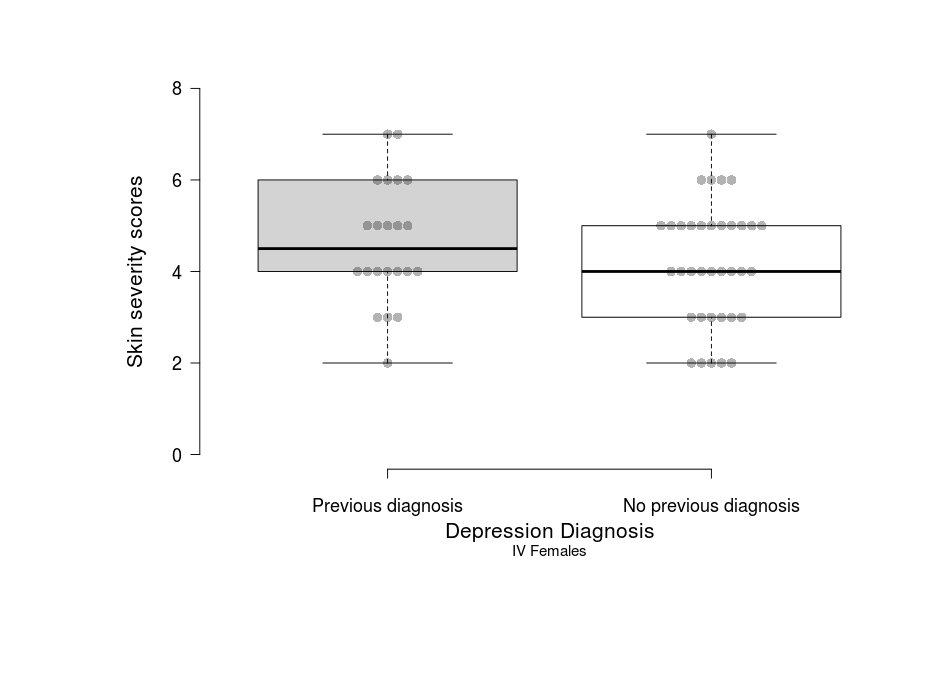

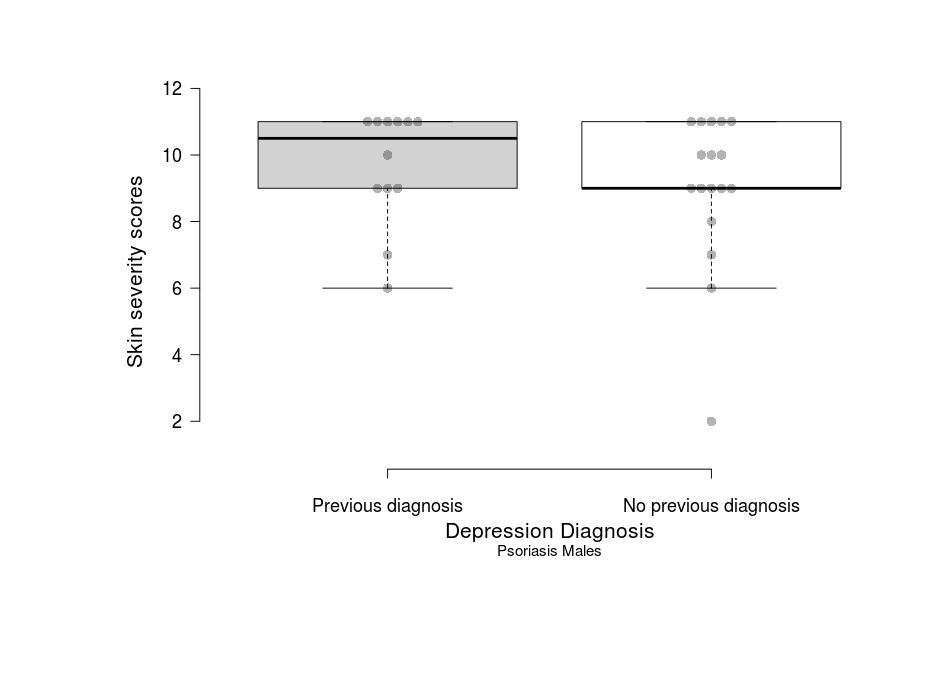


**D**

**C**


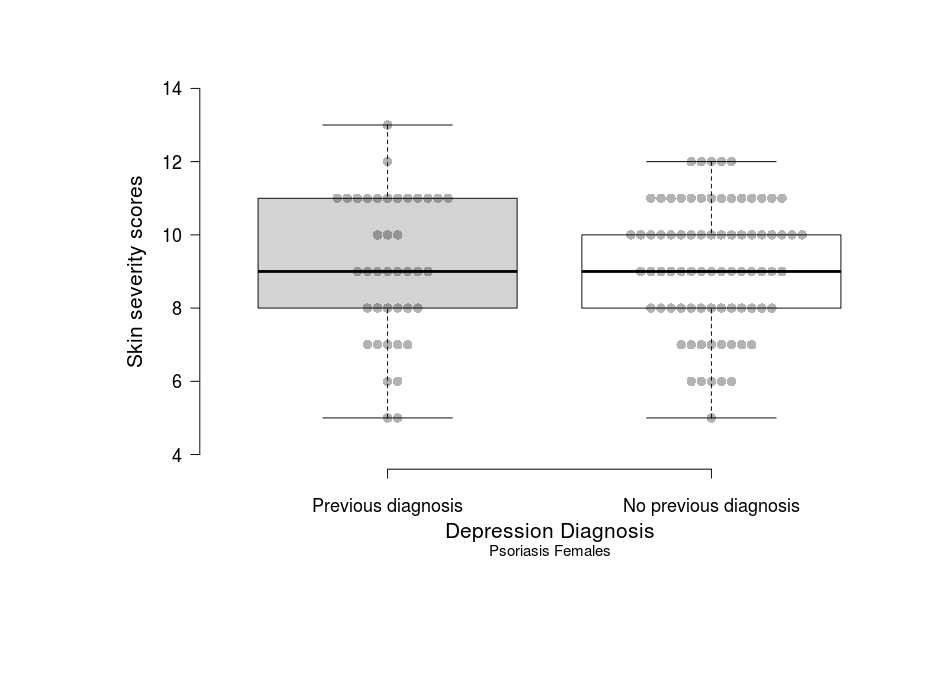


**E**

**Figure S2:** Ranking of factors (scored from 0-10) potentially predisposing to adult mood symptoms (depression (A)), anxiety (B) and irritability (C) in males with XLI

| **Rank** | **Mood Factor - Depression** |
| --- | --- |
| 1 | Moderate chronic life event (relationship difficulties/divorce, chronic illness, work-related stress etc.) |
| 2 | Stigma or bullying associated with your skin condition |
| 3 | Difficulties or frustration associated with treating your skin condition (e.g. regularly having to source and apply moisturizer) |
| 4 | Severe adverse life event (bereavement, life-threatening illness etc.) |
| 5 | Embarrassment of social interaction because of your skin condition |
| 6 | Sleep problems unrelated to your skin condition |
| 7 | Stigma or bullying due to finding it hard to pay attention e.g. getting distracted, not finishing work on time, not listening to instructions |
| 8 | Pain, discomfort or itching associated with your skin condition |
| 9 | Educational, work or social challenges due to finding it hard to pay attention e.g. getting distracted, not finishing work on time, not listening to instructions |
| 10 | Medical issues (non-skin related) |
| 11 | Low quality/quantity of friendships and relationships |
| 12 | Stigma or bullying due to difficulties with social interaction e.g. not understanding another person's point of view or social norms, finding it difficult to follow conversations. not understanding humour or sarcasm etc. |
| 13 | Sleep problems related to your skin condition e.g. due to excessive itchiness, night-time sweating etc. |
| 14 | Educational, work or social challenges due to being impulsive e.g. making rash decisions, interrupting when people are talking, doing things without thinking of the consequences etc. |
| 15 | Stigma or bullying due to being impulsive e.g. making rash decisions, interrupting when people are talking, doing things without thinking of the consequences etc. |
| 16 | Difficulty regulating body temperature/sweating |
| 17 | Educational, work or social challenges due to difficulties with social interaction e.g. not understanding another person's point of view or social norms, finding it difficult to follow conversations. not understanding humour or sarcasm etc. |
| 18 | Allergies |

**A**

**B**

| **Rank** | **Mood Factor - Anxiety** |
| --- | --- |
| 1 | Moderate chronic life event (relationship difficulties/divorce, chronic illness, work-related stress etc.) |
| 2 | Embarrassment of social interaction because of your skin condition |
| 3 | Stigma or bullying associated with your skin condition |
| 4 | Severe adverse life event (bereavement, life-threatening illness etc.) |
| 5 | Difficulties or frustration associated with treating your skin condition (e.g. regularly having to source and apply moisturizer) |
| 6 | Medical issues (non-skin related) |
| 7 | Educational, work or social challenges due to finding it hard to pay attention e.g. getting distracted, not finishing work on time, not listening to instructions |
| 8 | Sleep problems unrelated to your skin condition |
| 9 | Educational, work or social challenges due to being impulsive e.g. making rash decisions, interrupting when people are talking, doing things without thinking of the consequences etc. |
| 10 | Stigma or bullying due to finding it hard to pay attention e.g. getting distracted, not finishing work on time, not listening to instructions |
| 11 | Pain, discomfort or itching associated with your skin condition |
| 12 | Stigma or bullying due to being impulsive e.g. making rash decisions, interrupting when people are talking, doing things without thinking of the consequences etc. |
| 13 | Stigma or bullying due to difficulties with social interaction e.g. not understanding another person's point of view or social norms, finding it difficult to follow conversations. not understanding humour or sarcasm etc. |
| 14 | Sleep problems related to your skin condition e.g. due to excessive itchiness, night-time sweating etc. |
| 15 | Low quality/quantity of friendships and relationships |
| 16 | Difficulty regulating body temperature/sweating |
| 17 | Educational, work or social challenges due to difficulties with social interaction e.g. not understanding another person's point of view or social norms, finding it difficult to follow conversations. not understanding humour or sarcasm etc. |
| 18 | Allergies |

**C**

| **Rank** | **Mood Factor - Irritability** |
| --- | --- |
| 1 | Stigma or bullying associated with your skin condition |
| 2 | Moderate chronic life event (relationship difficulties/divorce, chronic illness, work-related stress etc.) |
| 3 | Difficulties or frustration associated with treating your skin condition (e.g. regularly having to source and apply moisturizer) |
| 4 | Embarrassment of social interaction because of your skin condition |
| 5 | Pain, discomfort or itching associated with your skin condition |
| 6 | Sleep problems unrelated to your skin condition |
| 7 | Severe adverse life event (bereavement, life-threatening illness etc.) |
| 8 | Stigma or bullying due to finding it hard to pay attention e.g. getting distracted, not finishing work on time, not listening to instructions |
| 9 | Sleep problems related to your skin condition e.g. due to excessive itchiness, night-time sweating etc. |
| 10 | Difficulty regulating body temperature/sweating |
| 11 | Educational, work or social challenges due to finding it hard to pay attention e.g. getting distracted, not finishing work on time, not listening to instructions |
| 12 | Stigma or bullying due to being impulsive e.g. making rash decisions, interrupting when people are talking, doing things without thinking of the consequences etc. |
| 13 | Stigma or bullying due to difficulties with social interaction e.g. not understanding another person's point of view or social norms, finding it difficult to follow conversations. not understanding humour or sarcasm etc. |
| 14 | Educational, work or social challenges due to being impulsive e.g. making rash decisions, interrupting when people are talking, doing things without thinking of the consequences etc. |
| 15 | Medical issues (non-skin related) |
| 16 | Educational, work or social challenges due to difficulties with social interaction e.g. not understanding another person's point of view or social norms, finding it difficult to follow conversations. not understanding humour or sarcasm etc. |
| 17 | Low quality/quantity of friendships and relationships |
| 18 | Allergies |

**Figure S3:** Ranking of factors (scored from 0-10) potentially predisposing to adult mood symptoms (depression (A)), anxiety (B) and irritability (C) in female XLI carriers

**A**

| **Rank** | **Mood Factor - Depression** |
| --- | --- |
| 1 | Moderate chronic life event (relationship difficulties/divorce, chronic illness, work-related stress etc.) |
| 2 | Severe adverse life event (bereavement, life-threatening illness etc.) |
| 3 | Sleep problems unrelated to your skin condition |
| 4 | Medical issues (non-skin related) |
| 5 | Low quality/quantity of friendships and relationships |
| 6 | Educational, work or social challenges due to finding it hard to pay attention e.g. getting distracted, not finishing work on time, not listening to instructions |
| 7 | Stress due to having a child with a long-term medical (skin) condition |
| 8 | Educational, work or social challenges due to difficulties with social interaction e.g. not understanding another person's point of view or social norms, finding it difficult to follow conversations. not understanding humour or sarcasm etc. |
| 9 | Stigma or bullying due to finding it hard to pay attention e.g. getting distracted, not finishing work on time, not listening to instructions |
| 10 | Educational, work or social challenges due to being impulsive e.g. making rash decisions, interrupting when people are talking, doing things without thinking of the consequences etc. |
| 11 | Stigma or bullying due to difficulties with social interaction e.g. not understanding another person's point of view or social norms, finding it difficult to follow conversations. not understanding humour or sarcasm etc. |
| 12 | Allergies |
| 13 | Stigma or bullying due to being impulsive e.g. making rash decisions, interrupting when people are talking, doing things without thinking of the consequences etc. |

**B**

| **Rank** | **Mood Factor - Anxiety** |
| --- | --- |
| 1 | Moderate chronic life event (relationship difficulties/divorce, chronic illness, work-related stress etc.) |
| 2 | Sleep problems unrelated to your skin condition |
| 3 | Severe adverse life event (bereavement, life-threatening illness etc.) |
| 4 | Medical issues (non-skin related) |
| 5 | Stress due to having a child with a long-term medical (skin) condition |
| 6 | Educational, work or social challenges due to finding it hard to pay attention e.g. getting distracted, not finishing work on time, not listening to instructions |
| 7 | Low quality/quantity of friendships and relationships |
| 8 | Educational, work or social challenges due to being impulsive e.g. making rash decisions, interrupting when people are talking, doing things without thinking of the consequences etc. |
| 9 | Educational, work or social challenges due to difficulties with social interaction e.g. not understanding another person's point of view or social norms, finding it difficult to follow conversations. not understanding humour or sarcasm etc. |
| 10 | Stigma or bullying due to finding it hard to pay attention e.g. getting distracted, not finishing work on time, not listening to instructions |
| 11 | Stigma or bullying due to difficulties with social interaction e.g. not understanding another person's point of view or social norms, finding it difficult to follow conversations. not understanding humour or sarcasm etc. |
| 12 | Stigma or bullying due to being impulsive e.g. making rash decisions, interrupting when people are talking, doing things without thinking of the consequences etc. |
| 13 | Allergies |

**C**

| **Rank** | **Mood Factor - Irritability** |
| --- | --- |
| 1 | Sleep problems unrelated to your skin condition |
| 2 | Moderate chronic life event (relationship difficulties/divorce, chronic illness, work-related stress etc.) |
| 3 | Medical issues (non-skin related) |
| 4 | Severe adverse life event (bereavement, life-threatening illness etc.) |
| 5 | Educational, work or social challenges due to finding it hard to pay attention e.g. getting distracted, not finishing work on time, not listening to instructions |
| 6 | Stress due to having a child with a long-term medical (skin) condition |
| 7 | Educational, work or social challenges due to difficulties with social interaction e.g. not understanding another person's point of view or social norms, finding it difficult to follow conversations. not understanding humour or sarcasm etc. |
| 8 | Educational, work or social challenges due to being impulsive e.g. making rash decisions, interrupting when people are talking, doing things without thinking of the consequences etc. |
| 9 | Low quality/quantity of friendships and relationships |
| 10 | Stigma or bullying due to finding it hard to pay attention e.g. getting distracted, not finishing work on time, not listening to instructions |
| 11 | Allergies |
| 12 | Stigma or bullying due to difficulties with social interaction e.g. not understanding another person's point of view or social norms, finding it difficult to follow conversations. not understanding humour or sarcasm etc. |
| 13 | Stigma or bullying due to being impulsive e.g. making rash decisions, interrupting when people are talking, doing things without thinking of the consequences etc. |

**Figure S4:** Ranking of factors (scored from 0-10) potentially predisposing to adult mood symptoms (depression (A)), anxiety (B) and irritability (C) in males with IV

**A**

| **Rank** | **Mood Factor - Depression** |
| --- | --- |
| 1 | Moderate chronic life event (relationship difficulties/divorce, chronic illness, work-related stress etc.) |
| 2 | Low quality/quantity of friendships and relationships |
| 3 | Embarrassment of social interaction because of your skin condition |
| 4 | Pain, discomfort or itching associated with your skin condition |
| 5 | Difficulties or frustration associated with treating your skin condition (e.g. regularly having to source and apply moisturizer) |
| 6 | Stigma or bullying associated with your skin condition |
| 7 | Sleep problems unrelated to your skin condition |
| 8 | Severe adverse life event (bereavement, life-threatening illness etc.) |
| 9 | Educational, work or social challenges due to finding it hard to pay attention e.g. getting distracted, not finishing work on time, not listening to instructions |
| 10 | Medical issues (non-skin related) |
| 11 | Stigma or bullying due to being impulsive e.g. making rash decisions, interrupting when people are talking, doing things without thinking of the consequences etc. |
| 12 | Stigma or bullying due to finding it hard to pay attention e.g. getting distracted, not finishing work on time, not listening to instructions |
| 13 | Educational, work or social challenges due to difficulties with social interaction e.g. not understanding another person's point of view or social norms, finding it difficult to follow conversations. not understanding humour or sarcasm etc. |
| 14 | Difficulty regulating body temperature/sweating |
| 15 | Educational, work or social challenges due to being impulsive e.g. making rash decisions, interrupting when people are talking, doing things without thinking of the consequences etc. |
| 16 | Stigma or bullying due to difficulties with social interaction e.g. not understanding another person's point of view or social norms, finding it difficult to follow conversations. not understanding humour or sarcasm etc. |
| 17 | Sleep problems related to your skin condition e.g. due to excessive itchiness, night-time sweating etc. |
| 18 | Allergies |

**B**

| **Rank** | **Mood Factor - Anxiety** |
| --- | --- |
| 1 | Moderate chronic life event (relationship difficulties/divorce, chronic illness, work-related stress etc.) |
| 2 | Pain, discomfort or itching associated with your skin condition |
| 3 | Difficulties or frustration associated with treating your skin condition (e.g. regularly having to source and apply moisturizer) |
| 4 | Severe adverse life event (bereavement, life-threatening illness etc.) |
| 5 | Embarrassment of social interaction because of your skin condition |
| 6 | Low quality/quantity of friendships and relationships |
| 7 | Educational, work or social challenges due to finding it hard to pay attention e.g. getting distracted, not finishing work on time, not listening to instructions |
| 8 | Stigma or bullying associated with your skin condition |
| 9 | Medical issues (non-skin related) |
| 10 | Sleep problems unrelated to your skin condition |
| 11 | Difficulty regulating body temperature/sweating |
| 12 | Stigma or bullying due to finding it hard to pay attention e.g. getting distracted, not finishing work on time, not listening to instructions |
| 13 | Sleep problems related to your skin condition e.g. due to excessive itchiness, night-time sweating etc. |
| 14 | Allergies |
| 15 | Stigma or bullying due to being impulsive e.g. making rash decisions, interrupting when people are talking, doing things without thinking of the consequences etc. |
| 16 | Educational, work or social challenges due to difficulties with social interaction e.g. not understanding another person's point of view or social norms, finding it difficult to follow conversations. not understanding humour or sarcasm etc. |
| 17 | Educational, work or social challenges due to being impulsive e.g. making rash decisions, interrupting when people are talking, doing things without thinking of the consequences etc. |
| 18 | Stigma or bullying due to difficulties with social interaction e.g. not understanding another person's point of view or social norms, finding it difficult to follow conversations. not understanding humour or sarcasm etc. |

| **Rank** | **Mood Factor - Irritability** |
| --- | --- |
| 1 | Moderate chronic life event (relationship difficulties/divorce, chronic illness, work-related stress etc.) |
| 2 | Pain, discomfort or itching associated with your skin condition |
| 3 | Difficulties or frustration associated with treating your skin condition (e.g. regularly having to source and apply moisturizer) |
| 4 | Stigma or bullying associated with your skin condition |
| 5 | Difficulty regulating body temperature/sweating |
| 6 | Low quality/quantity of friendships and relationships |
| 7 | Stigma or bullying due to being impulsive e.g. making rash decisions, interrupting when people are talking, doing things without thinking of the consequences etc. |
| 8 | Educational, work or social challenges due to difficulties with social interaction e.g. not understanding another person's point of view or social norms, finding it difficult to follow conversations. not understanding humour or sarcasm etc. |
| 9 | Sleep problems related to your skin condition e.g. due to excessive itchiness, night-time sweating etc. |
| 10 | Embarrassment of social interaction because of your skin condition |
| 11 | Medical issues (non-skin related) |
| 12 | Severe adverse life event (bereavement, life-threatening illness etc.) |
| 13 | Sleep problems unrelated to your skin condition |
| 14 | Stigma or bullying due to finding it hard to pay attention e.g. getting distracted, not finishing work on time, not listening to instructions |
| 15 | Educational, work or social challenges due to being impulsive e.g. making rash decisions, interrupting when people are talking, doing things without thinking of the consequences etc. |
| 16 | Educational, work or social challenges due to finding it hard to pay attention e.g. getting distracted, not finishing work on time, not listening to instructions |
| 17 | Stigma or bullying due to difficulties with social interaction e.g. not understanding another person's point of view or social norms, finding it difficult to follow conversations. not understanding humour or sarcasm etc. |
| 18 | Allergies |

**C**

**Figure S5:** Ranking of factors (scored from 0-10) potentially predisposing to adult mood symptoms (depression (A)), anxiety (B) and irritability (C) in females with IV

**A**

| **Rank** | **Mood Factor - Depression** |
| --- | --- |
| 1 | Difficulties or frustration associated with treating your skin condition (e.g. regularly having to source and apply moisturizer) |
| 2 | Stigma or bullying associated with your skin condition |
| 3 | Moderate chronic life event (relationship difficulties/divorce, chronic illness, work-related stress etc.) |
| 4 | Embarrassment of social interaction because of your skin condition |
| 5 | Severe adverse life event (bereavement, life-threatening illness etc.) |
| 6 | Pain, discomfort or itching associated with your skin condition |
| 7 | Educational, work or social challenges due to finding it hard to pay attention e.g. getting distracted, not finishing work on time, not listening to instructions |
| 8 | Difficulty regulating body temperature/sweating |
| 9 | Medical issues (non-skin related) |
| 10 | Sleep problems unrelated to your skin condition |
| 11 | Sleep problems related to your skin condition e.g. due to excessive itchiness, night-time sweating etc. |
| 12 | Allergies |
| 13 | Low quality/quantity of friendships and relationships |
| 14 | Educational, work or social challenges due to being impulsive e.g. making rash decisions, interrupting when people are talking, doing things without thinking of the consequences etc. |
| 15 | Stigma or bullying due to difficulties with social interaction e.g. not understanding another person's point of view or social norms, finding it difficult to follow conversations. not understanding humour or sarcasm etc. |
| 16 | Stigma or bullying due to being impulsive e.g. making rash decisions, interrupting when people are talking, doing things without thinking of the consequences etc. |
| 17 | Educational, work or social challenges due to difficulties with social interaction e.g. not understanding another person's point of view or social norms, finding it difficult to follow conversations. not understanding humour or sarcasm etc. |
| 18 | Stigma or bullying due to finding it hard to pay attention e.g. getting distracted, not finishing work on time, not listening to instructions |

| **Rank** | **Mood Factor - Anxiety** |
| --- | --- |
| 1 | Embarrassment of social interaction because of your skin condition |
| 2 | Stigma or bullying associated with your skin condition |
| 3 | Moderate chronic life event (relationship difficulties/divorce, chronic illness, work-related stress etc.) |
| 4 | Difficulties or frustration associated with treating your skin condition (e.g. regularly having to source and apply moisturizer) |
| 5 | Severe adverse life event (bereavement, life-threatening illness etc.) |
| 6 | Pain, discomfort or itching associated with your skin condition |
| 7 | Difficulty regulating body temperature/sweating |
| 8 | Medical issues (non-skin related) |
| 9 | Sleep problems unrelated to your skin condition |
| 10 | Allergies |
| 11 | Educational, work or social challenges due to finding it hard to pay attention e.g. getting distracted, not finishing work on time, not listening to instructions |
| 12 | Sleep problems related to your skin condition e.g. due to excessive itchiness, night-time sweating etc. |
| 13 | Educational, work or social challenges due to being impulsive e.g. making rash decisions, interrupting when people are talking, doing things without thinking of the consequences etc. |
| 14 | Low quality/quantity of friendships and relationships |
| 15 | Stigma or bullying due to difficulties with social interaction e.g. not understanding another person's point of view or social norms, finding it difficult to follow conversations. not understanding humour or sarcasm etc. |
| 16 | Stigma or bullying due to being impulsive e.g. making rash decisions, interrupting when people are talking, doing things without thinking of the consequences etc. |
| 17 | Educational, work or social challenges due to difficulties with social interaction e.g. not understanding another person's point of view or social norms, finding it difficult to follow conversations. not understanding humour or sarcasm etc. |
| 18 | Stigma or bullying due to finding it hard to pay attention e.g. getting distracted, not finishing work on time, not listening to instructions |

**B**

| **Rank** | **Mood Factor - Irritability** |
| --- | --- |
| 1 | Difficulties or frustration associated with treating your skin condition (e.g. regularly having to source and apply moisturizer) |
| 2 | Pain, discomfort or itching associated with your skin condition |
| 3 | Difficulty regulating body temperature/sweating |
| 4 | Moderate chronic life event (relationship difficulties/divorce, chronic illness, work-related stress etc.) |
| 5 | Sleep problems related to your skin condition e.g. due to excessive itchiness, night-time sweating etc. |
| 6 | Stigma or bullying associated with your skin condition |
| 7 | Embarrassment of social interaction because of your skin condition |
| 8 | Allergies |
| 9 | Medical issues (non-skin related) |
| 10 | Sleep problems unrelated to your skin condition |
| 11 | Educational, work or social challenges due to finding it hard to pay attention e.g. getting distracted, not finishing work on time, not listening to instructions |
| 12 | Severe adverse life event (bereavement, life-threatening illness etc.) |
| 13 | Educational, work or social challenges due to being impulsive e.g. making rash decisions, interrupting when people are talking, doing things without thinking of the consequences etc. |
| 14 | Low quality/quantity of friendships and relationships |
| 15 | Stigma or bullying due to difficulties with social interaction e.g. not understanding another person's point of view or social norms, finding it difficult to follow conversations. not understanding humour or sarcasm etc. |
| 16 | Stigma or bullying due to being impulsive e.g. making rash decisions, interrupting when people are talking, doing things without thinking of the consequences etc. |
| 17 | Stigma or bullying due to finding it hard to pay attention e.g. getting distracted, not finishing work on time, not listening to instructions |
| 18 | Educational, work or social challenges due to difficulties with social interaction e.g. not understanding another person's point of view or social norms, finding it difficult to follow conversations. not understanding humour or sarcasm etc. |

**C**

**Figure S6:** Ranking of factors (scored from 0-10) potentially predisposing to adult mood symptoms (depression (A)), anxiety (B) and irritability (C) in males with psoriasis

**A**

| **Rank** | **Mood Factor - Depression** |
| --- | --- |
| 1 | Moderate chronic life event (relationship difficulties/divorce, chronic illness, work-related stress etc.) |
| 2 | Pain, discomfort or itching associated with your skin condition |
| 3 | Difficulties or frustration associated with treating your skin condition (e.g. regularly having to source and apply moisturizer) |
| 4 | Severe adverse life event (bereavement, life-threatening illness etc.) |
| 5 | Sleep problems related to your skin condition e.g. due to excessive itchiness, night-time sweating etc. |
| 6 | Embarrassment of social interaction because of your skin condition |
| 7 | Difficulty regulating body temperature/sweating |
| 8 | Medical issues (non-skin related) |
| 9 | Low quality/quantity of friendships and relationships |
| 10 | Sleep problems unrelated to your skin condition |
| 11 | Stigma or bullying associated with your skin condition |
| 12 | Stigma or bullying due to being impulsive e.g. making rash decisions, interrupting when people are talking, doing things without thinking of the consequences etc. |
| 13 | Educational, work or social challenges due to finding it hard to pay attention e.g. getting distracted, not finishing work on time, not listening to instructions |
| 14 | Educational, work or social challenges due to difficulties with social interaction e.g. not understanding another person's point of view or social norms, finding it difficult to follow conversations. not understanding humour or sarcasm etc. |
| 15 | Allergies |
| 16 | Educational, work or social challenges due to being impulsive e.g. making rash decisions, interrupting when people are talking, doing things without thinking of the consequences etc. |
| 17 | Stigma or bullying due to difficulties with social interaction e.g. not understanding another person's point of view or social norms, finding it difficult to follow conversations. not understanding humour or sarcasm etc. |
| 18 | Stigma or bullying due to finding it hard to pay attention e.g. getting distracted, not finishing work on time, not listening to instructions |

**B**

| **Rank** | **Mood Factor - Anxiety** |
| --- | --- |
| 1 | Moderate chronic life event (relationship difficulties/divorce, chronic illness, work-related stress etc.) |
| 2 | Pain, discomfort or itching associated with your skin condition |
| 3 | Embarrassment of social interaction because of your skin condition |
| 4 | Medical issues (non-skin related) |
| 5 | Difficulties or frustration associated with treating your skin condition (e.g. regularly having to source and apply moisturizer) |
| 6 | Sleep problems related to your skin condition e.g. due to excessive itchiness, night-time sweating etc. |
| 7 | Severe adverse life event (bereavement, life-threatening illness etc.) |
| 8 | Sleep problems unrelated to your skin condition |
| 9 | Difficulty regulating body temperature/sweating |
| 10 | Educational, work or social challenges due to being impulsive e.g. making rash decisions, interrupting when people are talking, doing things without thinking of the consequences etc. |
| 11 | Stigma or bullying associated with your skin condition |
| 12 | Educational, work or social challenges due to difficulties with social interaction e.g. not understanding another person's point of view or social norms, finding it difficult to follow conversations. not understanding humour or sarcasm etc. |
| 13 | Educational, work or social challenges due to finding it hard to pay attention e.g. getting distracted, not finishing work on time, not listening to instructions |
| 14 | Low quality/quantity of friendships and relationships |
| 15 | Stigma or bullying due to being impulsive e.g. making rash decisions, interrupting when people are talking, doing things without thinking of the consequences etc. |
| 16 | Stigma or bullying due to finding it hard to pay attention e.g. getting distracted, not finishing work on time, not listening to instructions |
| 17 | Stigma or bullying due to difficulties with social interaction e.g. not understanding another person's point of view or social norms, finding it difficult to follow conversations. not understanding humour or sarcasm etc. |
| 18 | Allergies |

**C**

| **Rank** | **Mood Factor - Irritability** |
| --- | --- |
| 1 | Difficulties or frustration associated with treating your skin condition (e.g. regularly having to source and apply moisturizer) |
| 2 | Pain, discomfort or itching associated with your skin condition |
| 3 | Sleep problems related to your skin condition e.g. due to excessive itchiness, night-time sweating etc. |
| 4 | Moderate chronic life event (relationship difficulties/divorce, chronic illness, work-related stress etc.) |
| 5 | Difficulty regulating body temperature/sweating |
| 6 | Embarrassment of social interaction because of your skin condition |
| 7 | Severe adverse life event (bereavement, life-threatening illness etc.) |
| 8 | Medical issues (non-skin related) |
| 9 | Educational, work or social challenges due to finding it hard to pay attention e.g. getting distracted, not finishing work on time, not listening to instructions |
| 10 | Sleep problems unrelated to your skin condition |
| 11 | Allergies |
| 12 | Stigma or bullying associated with your skin condition |
| 13 | Educational, work or social challenges due to difficulties with social interaction e.g. not understanding another person's point of view or social norms, finding it difficult to follow conversations. not understanding humour or sarcasm etc. |
| 14 | Stigma or bullying due to finding it hard to pay attention e.g. getting distracted, not finishing work on time, not listening to instructions |
| 15 | Educational, work or social challenges due to being impulsive e.g. making rash decisions, interrupting when people are talking, doing things without thinking of the consequences etc. |
| 16 | Low quality/quantity of friendships and relationships |
| 17 | Stigma or bullying due to being impulsive e.g. making rash decisions, interrupting when people are talking, doing things without thinking of the consequences etc. |
| 18 | Stigma or bullying due to difficulties with social interaction e.g. not understanding another person's point of view or social norms, finding it difficult to follow conversations. not understanding humour or sarcasm etc. |

**Figure S7:** Ranking of factors (scored from 0-10) potentially predisposing to adult mood symptoms (depression (A)), anxiety (B) and irritability (C) in females with psoriasis

**A**

| **Rank** | **Mood Factor - Depression** |
| --- | --- |
| 1 | Pain, discomfort or itching associated with your skin condition |
| 2 | Moderate chronic life event (relationship difficulties/divorce, chronic illness, work-related stress etc.) |
| 3 | Difficulties or frustration associated with treating your skin condition (e.g. regularly having to source and apply moisturizer) |
| 4 | Embarrassment of social interaction because of your skin condition |
| 5 | Severe adverse life event (bereavement, life-threatening illness etc.) |
| 6 | Sleep problems related to your skin condition e.g. due to excessive itchiness, night-time sweating etc. |
| 7 | Sleep problems unrelated to your skin condition |
| 8 | Medical issues (non-skin related) |
| 9 | Stigma or bullying associated with your skin condition |
| 10 | Low quality/quantity of friendships and relationships |
| 11 | Educational, work or social challenges due to finding it hard to pay attention e.g. getting distracted, not finishing work on time, not listening to instructions |
| 12 | Educational, work or social challenges due to being impulsive e.g. making rash decisions, interrupting when people are talking, doing things without thinking of the consequences etc. |
| 13 | Difficulty regulating body temperature/sweating |
| 14 | Stigma or bullying due to finding it hard to pay attention e.g. getting distracted, not finishing work on time, not listening to instructions |
| 15 | Educational, work or social challenges due to difficulties with social interaction e.g. not understanding another person's point of view or social norms, finding it difficult to follow conversations. not understanding humour or sarcasm etc. |
| 16 | Stigma or bullying due to being impulsive e.g. making rash decisions, interrupting when people are talking, doing things without thinking of the consequences etc. |
| 17 | Stigma or bullying due to difficulties with social interaction e.g. not understanding another person's point of view or social norms, finding it difficult to follow conversations. not understanding humour or sarcasm etc. |
| 18 | Allergies |

**B**

| **Rank** | **Mood Factor - Anxiety** |
| --- | --- |
| 1 | Moderate chronic life event (relationship difficulties/divorce, chronic illness, work-related stress etc.) |
| 2 | Severe adverse life event (bereavement, life-threatening illness etc.) |
| 3 | Pain, discomfort or itching associated with your skin condition |
| 4 | Sleep problems related to your skin condition e.g. due to excessive itchiness, night-time sweating etc. |
| 5 | Embarrassment of social interaction because of your skin condition |
| 6 | Difficulties or frustration associated with treating your skin condition (e.g. regularly having to source and apply moisturizer) |
| 7 | Sleep problems unrelated to your skin condition |
| 8 | Medical issues (non-skin related) |
| 9 | Stigma or bullying associated with your skin condition |
| 10 | Educational, work or social challenges due to finding it hard to pay attention e.g. getting distracted, not finishing work on time, not listening to instructions |
| 11 | Low quality/quantity of friendships and relationships |
| 12 | Difficulty regulating body temperature/sweating |
| 13 | Educational, work or social challenges due to being impulsive e.g. making rash decisions, interrupting when people are talking, doing things without thinking of the consequences etc. |
| 14 | Stigma or bullying due to finding it hard to pay attention e.g. getting distracted, not finishing work on time, not listening to instructions |
| 15 | Educational, work or social challenges due to difficulties with social interaction e.g. not understanding another person's point of view or social norms, finding it difficult to follow conversations. not understanding humour or sarcasm etc. |
| 16 | Stigma or bullying due to being impulsive e.g. making rash decisions, interrupting when people are talking, doing things without thinking of the consequences etc. |
| 17 | Stigma or bullying due to difficulties with social interaction e.g. not understanding another person's point of view or social norms, finding it difficult to follow conversations. not understanding humour or sarcasm etc. |
| 18 | Allergies |

| **Rank** | **Mood Factor - Irritability** |
| --- | --- |
| 1 | Pain, discomfort or itching associated with your skin condition |
| 2 | Sleep problems related to your skin condition e.g. due to excessive itchiness, night-time sweating etc. |
| 3 | Difficulties or frustration associated with treating your skin condition (e.g. regularly having to source and apply moisturizer) |
| 4 | Sleep problems unrelated to your skin condition |
| 5 | Moderate chronic life event (relationship difficulties/divorce, chronic illness, work-related stress etc.) |
| 6 | Embarrassment of social interaction because of your skin condition |
| 7 | Difficulty regulating body temperature/sweating |
| 8 | Severe adverse life event (bereavement, life-threatening illness etc.) |
| 9 | Educational, work or social challenges due to finding it hard to pay attention e.g. getting distracted, not finishing work on time, not listening to instructions |
| 10 | Medical issues (non-skin related) |
| 11 | Stigma or bullying associated with your skin condition |
| 12 | Low quality/quantity of friendships and relationships |
| 13 | Educational, work or social challenges due to being impulsive e.g. making rash decisions, interrupting when people are talking, doing things without thinking of the consequences etc. |
| 14 | Allergies |
| 15 | Educational, work or social challenges due to difficulties with social interaction e.g. not understanding another person's point of view or social norms, finding it difficult to follow conversations. not understanding humour or sarcasm etc. |
| 16 | Stigma or bullying due to finding it hard to pay attention e.g. getting distracted, not finishing work on time, not listening to instructions |
| 17 | Stigma or bullying due to being impulsive e.g. making rash decisions, interrupting when people are talking, doing things without thinking of the consequences etc. |
| 18 | Stigma or bullying due to difficulties with social interaction e.g. not understanding another person's point of view or social norms, finding it difficult to follow conversations. not understanding humour or sarcasm etc. |

**C**

**Table S6.** A qualitative deductive content analysis of free-text comments relating to factors underlying mood symptoms across the lifespan across the three conditions. Inter-rater reliability across two coders was assessed by Cohen’s κ and indicated substantial and significant agreement (κ=0.73, (95% CI: 0.65-0.80), p<0.001).

**Table S7.** Internal consistency as indexed by Cronbach’s alpha (α) across questionnaires and across experimental groups.

| **Group** | **Questionnaire** | | | | | |
| --- | --- | --- | --- | --- | --- | --- |
|  | **K10** | **AQ10** | **ASRS** | **BITE** | **DLQI** | **FSQ** |
| **XLI males** | 0.94 | 0.61 | 0.90 | 0.94 | 0.85 | 0.93 |
| **XLI females** | 0.89 | 0.72 | 0.92 | 0.91 | - | - |
| **IV males** | 0.78 | 0.60 | 0.80 | 0.94 | 0.87 | 0.95 |
| **IV females** | 0.89 | 0.56 | 0.87 | 0.89 | 0.76 | 0.89 |
| **Psoriasis males** | 0.90 | 0.66 | 0.91 | 0.93 | 0.84 | 0.90 |
| **Psoriasis females** | 0.90 | 0.69 | 0.90 | 0.92 | 0.84 | 0.90 |
| **Mean α** | 0.90 | 0.67 | 0.90 | 0.92 | 0.84 | 0.90 |
